# The Physiology of Cognition in Autism Spectrum Disorder: Current and Future Challenges

**DOI:** 10.7759/cureus.46581

**Published:** 2023-10-06

**Authors:** Sarah H Al-Mazidi

**Affiliations:** 1 Physiology, Imam Mohammad Ibn Saud Islamic University, Riyadh, SAU

**Keywords:** physiology, asd, diagnosis, cognition, autism spectrum disorder

## Abstract

Cognitive impairment is among the most challenging characteristics of autism spectrum disorder (ASD). Although ASD is one of the common neurodevelopmental disorders, we are still behind in diagnosing and treating cognitive impairment in ASD. Cognitive impairment in ASD varies, meaning it could be at the sensory perception level to cognitive processing, learning, and memory. There are no diagnostic criteria for cognitive impairment that are specific to ASD. The leading causes of cognitive impairment in ASD could be neurological, immune, and gastrointestinal dysfunction. Immune dysfunction might lead to neuroinflammation, affecting neural connectivity, glutamate/gamma-aminobutyric acid (GABA) balance, and plasticity. The gut-brain axes are essential in the developing brain. Special retinal changes have recently been detected in ASD, which need clinical investigation to find their possible role in early diagnosis. Early intervention is crucial for ASD cognitive dysfunction. Due to the heterogeneity of the disease, the clinical manifestation of ASD makes it difficult for clinicians to develop gold-standard diagnostic and therapeutic criteria. We suggest a triad for diagnosis, which includes clinical tests for immune and gastrointestinal dysfunction biomarkers, clinical examination for the retina, and an objective neurocognitive evaluation for ASD, and to develop a treatment strategy involving these three aspects. Developing clear treatment criteria for cognitive impairment for ASD would improve the quality of life of ASD people and their caregivers and would delay or prevent dementia-related disorders in ASD people.

## Introduction and background

Autism spectrum disorder (ASD) is one of the most common neurodevelopmental disorders. The diagnosis of ASD is based on behavioral characteristics and cognitive functions, which are usually the first signs parents notice to seek medical attention. Although cognitive dysfunction is not associated with all ASD people, it is commonly mistaken with other intellectual disabilities and attention deficit hyperactive disorders [1]. ASD people who do not show cognitive impairment have social cognition and cognitive inflexibility, making dealing with altered daily situations challenging for them [2].

The physiological aspect of ASD correlates systemic functions to behaviors of ASD, which are repetitive, restricted behaviors, and impaired social/communication skills. The systems affected by ASD and related to cognitive dysfunction are the neural, immune, and gastrointestinal systems. Many biomarkers have been proposed to indicate neural, immune, and gastrointestinal dysfunction. None of the biomarkers has been used for diagnostic or therapeutic purposes because of the heterogeneity of ASD clinical manifestation and the lack of clinical trials for these biomarkers.

This review focuses on determining the physiology of cognitive dysfunction in ASD and the possible link between cognitive dysfunction and biomarkers available in the literature. This will open a new research venue for gold-standard diagnostic and therapeutic criteria for cognitive impairment in ASD.

## Review

Assessment of cognitive function in ASD

There is no specific test for the clinical features of ASD. Therefore, the beginning of the diagnosis process depends on the caregiver's or teachers' observation of the child's behavior before seeking medical attention. The diagnosis can be made as early as two years of life. However, it is usually made by the age of three when the child is late in speaking and develops difficulties in socializing and communicating with his peers. At this age, some of the ASD children are misdiagnosed with intellectual disabilities.

There are specific tests to diagnose ASD and to differentiate between ASD and other intellectual disabilities. The most used and valid tests are the Childhood Autism Rating Scale (CARS) and the Autism Diagnostic Observation Schedule (ADOS). These scales are based on observations from trained healthcare personnel, usually a psychologist, and can be used as early as two years of age.

**Figure 1:**
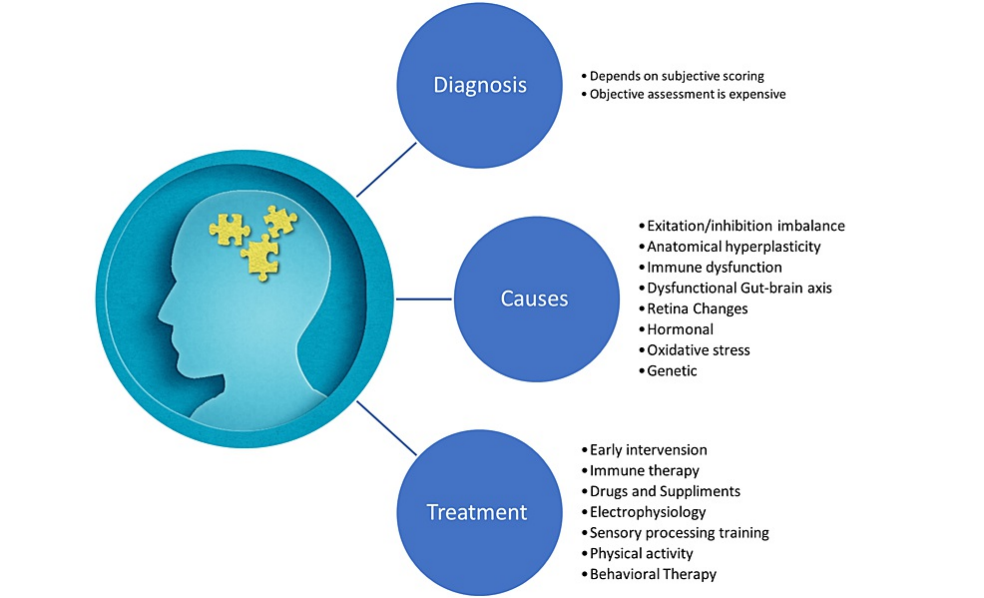
Current cognitive impairment diagnosis and intervention in autism spectrum disorder. Image created by the author.

The neurocognitive impairments associated with ASD are executive and social cognition dysfunction. After the child is diagnosed with ASD, multiple validated tests can determine the cognitive and intellectual functions. These tests are performed by trained personnel who rate the patient's intellectual abilities by a specific scoring system; the most popular tests are the Differential Ability Scales, 2nd Edition (DAS-II), which test the intelligence quotient (IQ) and the Wechsler Intelligence Scale for Children - 5th Edition (WISC-V), which evaluates the IQ as well. Also, an objective tool that evaluates working memory through specific domains is the Cambridge Neuropsychological Test Automated Battery (CANTAB®) [2]. Many of these scales should be used cautiously because of the discrepancies in the severity and etiology of cognitive dysfunction in ASD people [3,4]. For example, the WISC-V was designed for neurotypical individuals. Therefore, the results of this scale were much higher in the non-verbal task than in verbal tasks in ASD, giving a different cognitive profile for each ASD person [4]. Similar results were found in non-verbal tasks of DAS-II [3]. Also, these tests did not adequately evaluate cognitive impairment related to social cognition, which is the primary type of cognitive dysfunction in ASD people [5]. The information that will be discussed in this review is shown in Figure 1.

Brief physiology of cognition

The cognitive functions refer to memory, learning, perception, and processing. It also includes language and behavior. In psychology, cognitive functions are affected by motivation and environmental factors. In physiology, many other factors affect cognitive function, including neural processing (pre and post-synaptic), hormones, and the immune system. Blood gases, glucose levels, and oxidative stress can also interfere with the physiology of cognition [6]. In addition, research found that cognition can be affected at the cellular and genetic levels [7-9].

The cognitive functions require optimal arousal and attention to perceive and react to changing environmental conditions by sensory perception (visual, auditory, and tactile). Specifically, vision has been associated with learning, memory, and decision-making [6]. Failure of any part of the sensory perception and integration would also affect behavior, emotions, and cognition. Vision is the most powerful sensation. A vision or visual perception defect might affect the auditory and tactile modalities. Neuroimaging-based findings reported that the perception of vision, auditory, and tactile sensations are integrated, meaning that we need all three modalities to be at their optimal function to perceive information and for learning to occur [10]. The perception of these sensations should be at the physiological level. Any disorder that leads to underactive or hyperactive sensory perception would affect cognitive processing [11].

Normal cortical excitability and connectivity are crucial for cognitive processing. This process requires multiple synapses and neurotransmitters for learning and plasticity to occur. These neurotransmitters and synapses essential for plasticity include gamma-aminobutyric acid (GABA), glutamate, acetylcholine, and dopamine [8,9]. Glutamine (excitatory) and GABA (inhibitory) ratios are balanced in normal individuals. The glutamine/GABA imbalance leads to cognitive impairment, especially in the developing brain [12]. Glutamine excites neurons leading to calcium influx and action potential. This occurs during learning and plasticity (potentiation) and is inhibited by GABA. Both neurotransmitters must be balanced for learning, especially during the developmental stage [9,12]. Over-excitation of glutamate leads to overstimulation of neurons and neural death in a process known as excitotoxicity.

Another concept in cognition is cognitive reserve. Cognitive reserve is a neuroprotective concept that is the process of preserving individual lifetime intellectual activities which is a vital determinant in brain aging and recovery from brain damage [13]. Over the years, the brain develops learning and compensatory processes to protect all cognitive domains. It is a predictor of cognitive performance, and it depends on multiple factors, including the intelligence of a person, learning experience, and environmental factors [14]. However, studies are required to validate the measuring tools for cognitive reserve.

Because cognition is affected in multiple physiological axes, neurological disorders involving cognitive impairment might share etiologies that might aid in understanding similar disorders. However, individuals with ASD might manifest different levels of cognitive impairments with different etiologies.

Brain abnormalities in ASD cognition

In ASD, there are different types and severity of cognitive impairment, which might differ between ASD people. Therefore, cognitive functions in ASD are affected at both physiological and psychological levels. Neuroinflammation, oxidative stress, and gut-brain axis are reported to be dysfunctional in ASD people, affecting cognitive physiology, which makes the therapeutic strategies for cognitive impairment challenging.

Brain structure and volume abnormalities were reported in ASD people in middle childhood [15,16]. However, recent studies reported that brain size does not affect cognitive functions [15,17]. In the early years of development, there is a significant increase in cortical and cerebellar white matter, which declines in late childhood and adolescence compared to neurotypical children [16,18]. Some areas of the cortex have overconnectivity, while others have underconnectivity [19]. The anatomical changes observed in ASD children affect the neural excitation/inhibition balance associated with ASD symptoms, such as social behaviors [20]. Stimulation of the cortex in certain areas modulates the GABAergic and glutaminergic neurons have improved social cognition in ASD people [2].

The function of these neurons is also affected by factors other than their anatomy. Studies also found that oxidative stress and low antioxidants are associated with cognitive impairment and abnormal behavior. Free radicals and antioxidants have a significant physiological role in the immune defense mechanism. They also have a major role in cell growth, intercellular communication, and defense against bacteria and viruses [21-23]. Therefore, oxidative stress affects cognitive function by directly damaging neurons and indirectly through the immune system leading to neuroinflammation.

Cognitive reserve, which determines cognitive performance in older age, might be dysfunctional in older ASD people. A study reported that ASD people are 2.6 times more likely to have dementia than typical older adults [24]. This was supported by a striking finding that the mean age of death in ASD people was lower than the general population, and the cause of death of 6% of ASD people was dementia-related disorders [25]. An older study reported a different result. They speculate that hyperplasticity occurs in the anatomically large white matter in ASD people and has a protective barrier against age-related dementia [26]. Age-related dementia has been reported in ASD with great discrepancies, which requires well-designed large-scale research, including molecular and experimental aspects. Also, an early diagnosis of ASD cognitive functions is crucial to ensure a better quality of life and to delay dementia and its related disorders.

Flexible cognition is the ability to change behavior and thoughts according to changing environmental situations. Neuroimaging studies showed that cognitive flexibility requires the activation of multiple cortical areas that interconnect to decide the adjusted behavior [27,28]. These connections need normal cortical excitability and balance in neurotransmitters such as GABA and glutamate [29]. Neuroimaging and neurocognitive tests showed that cognitive flexibility is impaired in ASD, which becomes more deficient with increased severity of ASD [28,30,31]. An interesting finding is that cognitive and behavioral flexibility are affected by oxytocin and vasopressin, and it is a suggested pharmacological treatment for cognitive flexibility in ASD [32]. Oxytocin and vasopressin are dysregulated in ASD people [33]. Besides its function in the reproductive system, oxytocin has many neural effects in the central nervous system, especially the hypothalamus and amygdala. It also affects neurons' excitation/inhibition balance and is associated with learning and memory [34]. Cognition flexibility is needed in other cognitive development, such as transitioning to adulthood. It is well known that ASD children transitioning into adulthood are delayed [35]. Therefore, we speculate that impaired cognitive flexibility is one of the major factors that delay ASD children transitioning into adulthood and one of the reasons for cognitive decline in aging ASD adults. It might also be a result of cortical excitation/inhibition imbalance observed in ASD.

The cognitive impairment in ASD people might be caused by sensory perception, especially visual processing [36]. The retina is an extension of the central nervous system, sharing an anatomical and embryological origin. The optic nerve originates from the brain. The eye also shares immune cells and vasculature similar to the central nervous system [37]. So, it is logical that neurodegenerative diseases affect the retina and can be diagnosed through the eye. Much research has connected the retina to cognitive impairment in neurodegenerative diseases such as Alzheimer's disease and correlated the electrophysiological and anatomical changes of the retina to the severity of cognitive diseases [38]. These tests, including electroretinogram (ERG) and optical coherence tomography (OCT), are noninvasive and available [37]. Studies have reported that fundus imaging can diagnose ASD [39,40]. Autistic people are hypersensitive to certain sensations, such as loud noises and bright lights. They also seek tactile modalities, referred to as proprioceptive seeking, which counts as one of the behavioral symptoms of ASD. Vision is the most powerful sensation [11]. We speculate that abnormalities in vision or visual recognition might have affected the other sensations (tactile and auditory), leading to behavioral and cognitive impairment. Retina examination might aid in innovating a vision-based therapy that might improve cognitive functions in ASD. With this growing evidence of the correlation between specific eye changes and ASD, can the eye be the definite clinical diagnostic criteria and a severity indicator for ASD?

Systemic abnormalities in ASD cognition

Although there are structural changes in the cortex of ASD patients, the cognitive dysfunction that is found in autistic people might originate from other source than the central nervous system [41]. Dysfunctions in the immune and gastrointestinal (GI) systems are reported in ASD. These include food allergy-related and non-food allergy-related. An imbalance in the interleukins (IL) was reported in the ASD cytokine profile. For example, increased inflammatory cytokines (IL-1 and IL-6) and reduced anti-inflammatory cytokines (IL-10) were found to be correlated with ASD behavioral symptoms [42]. Other inflammatory dysregulation in ASD and associated with cognitive impairment are interferon-gamma, tumor necrosis factor-alpha, and growth factors [7,43]. The gut microbiota is dysregulated in ASD, which is believed to affect the development of the central nervous system and the immune responses, referred to as the gut-brain axis.

Normal immune regulation is required for the physiological development of the brain. Many immune molecules are expressed in the developing brain, including cytokines, toll-like receptors, and growth factors, which modulate the brain's development and cognitive function [44]. Dysregulation of the immune system leads to neuroinflammation. There is mounting evidence that biomarkers of neuroinflammation are detected in ASD. Neuroinflammation affects synapsis, neuron function, connectivity, plasticity, cognitive processing, memory, and learning [45,46]. However, many treatments for immune dysfunction, food allergy, and chronic inflammation are ineffective in treating cognitive impairment in ASD or did not continue as a clinical trial [42].

Oxidative stress, dysfunctional mitochondria, and heavy metals were connected to cognitive impairment. Antioxidants are crucial for the developing brain, especially neural connections and plasticity. Learning and memory in neurodegenerative diseases are affected by oxidative stress, so cognitive function in ASD might also be affected by oxidative stress and mitochondria dysfunction [47-50]. Oxidative stress and cytokines are pathological factors affecting the myelination of neurons in ASD. Many studies reported that both free radicals and abnormal myelination were found in animal models of ASD and neuroimaging in ASD people. However, the literature did not conclude if the cognitive impairment caused by free radicals directly affects neurons' myelination or if they act indirectly by the immune system leading to neuroinflammation, GI dysregulation, or chronic inflammation. Extensive research has been done in this area relating anatomical, molecular, genetic, and physiological abnormalities in ASD people. However, none developed a diagnostic or therapeutic strategy for cognitive impairment in ASD people.

Many biomarkers of neuroinflammation and cognitive impairments were identified in ASD. Some of these biomarkers are shared among cognitive impairment in neurodegenerative and neurodevelopmental diseases. However, there is no specific biomarker for cognitive impairment in ASD. Genetic biomarkers were also associated with cognitive dysfunction in ASD, but because of the heterogeneity of the disease, it is not a valid tool for diagnosing cognitive impairment [51]. There is still a need for physiological, molecular, and genetic studies elucidating the pathophysiology of cognitive impairment in ASD [41].

Possible therapeutics for cognitive function in ASD

Because of the heterogeneity of the clinical presentation of ASD people and the lack of biomarkers specifically for ASD, there is a diversity of treatment plans and therapeutic techniques among ASD centers. Some parents resort to complementary and alternative medicine, desperately trying to find a cure. Currently, there are no effective pharmaceutical treatments.

Many cognitive and behavioral training techniques, such as cognitive remediation and cognitive enhancement therapy, are innovative and used by psychologists and psychiatrists in cognitive disorders such as schizophrenia that can also be used in ASD [52]. Behavioral therapy is one of the most effective therapies for ASD and one of the parents' favorite interventions [53]. Occupational therapy and physical activities effectively improve sensory integration because they affect sensory perception and increase serotonin levels, improving cognitive and general mental well-being [54].

Nutrition therapy is one of the effective therapies for ASD. Reducing gluten and increasing fiber in diet improved cognition and behavior in ASD people. Also, an alternating variety of food improved sensory perception and cognitive function in ASD children [55]. Prebiotic supplementation and microbiota restoration improve the beneficial bacteria in the digestive system and ameliorate cognitive dysfunction [56].

Pharmacological treatments that are commonly used for ASD are to treat certain associated systemic or psychiatric disorders. Pharmacological and dietary supplementation such as vitamin D, vitamin C, omega 3, sulforaphane, melatonin, and oxytocin were proposed in several studies and found to be effective in the cognitive function of ASD [57]. Antioxidant supplementation was beneficial in many ASD studies. However, because of the heterogeneity of ASD clinical manifestations, results are inconclusive and cannot be applied as a definite therapy for cognitive impairment in ASD [48]. Also, immune therapy studies showed promising results in ASD children with immune dysfunction, improving their behavioral symptoms and cognitive functions [57]. However, there is still a need for randomized clinical trials to validate the effectiveness of these drugs and supplements.

Electrophysiology was used in research to find the effect of electrical stimulation of cortical regions on the cognitive and behavioral outcomes of ASD people. Using the transcranial direct current stimulation to stimulate the prefrontal area and modulate cortical excitability noninvasively has improved cognitive processing in ASD people [2].

Because of mounting evidence of the structural changes in ASD people, an ultimate cure, in our opinion, is not possible. However, innovative therapies can target cognitive impairment in ASD people to improve their quality of life.

Suggestions

Figure 1 shows a diagram of the flow of information discussed in this review. First, we discussed the diagnostic tools for cognitive impairment, which are subjective depending on the personal rating of trained personnel. CANTAB is an objective tool for cognitive impairment, but it is costly and unavailable in clinics and primary care centers. There is an urgent need for an objective diagnostic tool for cognitive impairment specific to ASD. Then we discussed the possible causes and risk factors of cognitive impairment in ASD. Because of the heterogeneity of ASD, many factors contribute to cognitive impairment. The anatomical changes in volume and plasticity affect cortical excitability and might serve as a neuroprotective mechanism against dementia in older people. The gut, immune, and brain axis are reported in many clinical studies and reached saturation in beneficial evidence on cognitive and behavioral symptoms in ASD. It requires serious attention for randomized clinical trials.

**Figure 2:**
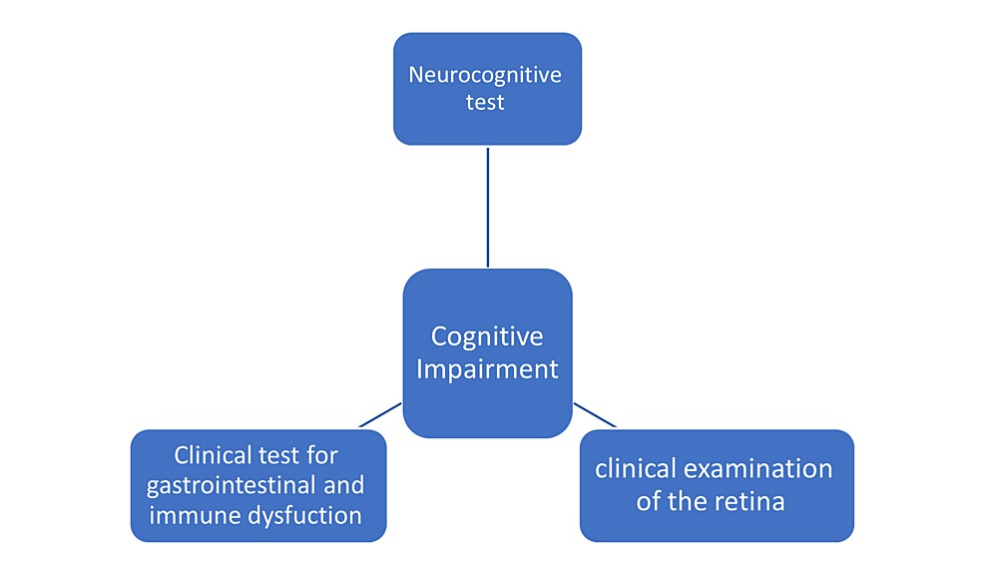
Suggested triad for diagnosis of cognitive impairment in autism spectrum disorder. Image created by the author.

This review represents the beginning of developing a new approach for diagnosing and treating cognitive impairment in ASD. As discussed in this review, cognitive dysfunction in ASD results from multiple systemic disorders reflected as behaviors. Therefore, it is time for randomized clinical trials targeting immune and GI systems that should also be integrated into a gold standard diagnosis and therapy for ASD with respect to the individual variation (Figure 2). Also, retinal changes were extensively studied in many cognitive disorders. However, research involving retina dysfunction and its relation to cognitive impairment in ASD is scarce, and it can be an objective diagnostic procedure for ASD diagnosis and a new venue for future research.

## Conclusions

The increased population of ASD people with cognitive dysfunction adds urgency to develop early diagnosis and intervention criteria for cognitive impairment designed especially for ASD. The heterogeneity of cognition in ASD allows innovative therapeutic techniques to be developed and appropriately used for each case. Early intervention is the most effective therapy; therefore, finding biomarkers is crucial for early diagnosis intervention.
